# Rapid, effective and user-friendly immunophenotyping of canine lymphoma using a personal flow cytometer

**DOI:** 10.1186/2046-0481-66-6

**Published:** 2013-04-01

**Authors:** Stratos Papakonstantinou, Inese Berzina, Amanda Lawlor, Emma J O’Neill, Peter J O’Brien

**Affiliations:** 1 School of Veterinary Medicine, University College Dublin (UCD), Dublin, Ireland

**Keywords:** Immunophenotyping, Canine lymphoma, Personal flow cytometer, Microfluidics, Guava

## Abstract

**Background:**

Widespread use of flow cytometry for immunophenotyping in clinical veterinary medicine is limited by cost and requirement for considerable laboratory space, staff time, and expertise. The Guava EasyCyte Plus (Guava Technologies, Hayward, CA, US) is the first, personal, bench-top flow cytometer designed to address these limitations.

**Objective:**

The aim of this study was to adapt the immunohistochemical protocol used for immunophenotyping of canine lymphoma to the personal flow cytometer for rapid, effective and user-friendly application to the diagnosis and prognosis of canine lymphoma and to demonstrate its practicality for widespread veterinary application. Performance of the personal flow cytometer for immunophenotyping T and B lymphocytes in blood and lymph nodes from normal dogs and dogs with lymphoproliferative disease, was assessed using only two monoclonal antibodies (against CD3 and CD21), and by comparison with analysis using two conventional flow cytometers.

**Methods:**

26 dogs with lymphoproliferative disease (23 with lymphoma, 3 with lymphocytic leukaemia) were studied along with 15 controls (2 non-lymphoma lymph nodes and 13 non-leukemic bloods. Lymphocytes were immunostained with fluorescent-labeled, monoclonal antibodies against CD3 and CD21. To assess the effectiveness of the personal flow cytometer in discrimination between T and B cell immunophenotypes, T and B cell counts for half the samples (14 blood and 11 lymph node) were also determined using the same method and conventional flow cytometers (FACSCalibur, Cyan Dako). To assess the effectiveness of the personal flow cytometer in discriminating between leukocyte types, lymphocyte differential counts were determined for 21 blood samples and compared with those from automated hematology analyzers (CELL-DYN 3500, n=11 and ADVIA 2120, n=10). Quality and sub-cellular distribution of immunostaining was assessed using fluorescence microscopy.

**Results:**

The protocol for immunophenotyping took 2 to 3 hours to complete from the point of receipt of sample to reporting of immunophenotype. The personal flow cytometer differential lymphocyte counts correlated highly (n=20; r=0.97, p<0.0001) with those of automated haematology analyzers. The personal flow cytometer counts consistently, but mildly, underestimated the percentages of lymphocytes in the samples (mean bias of -5.3%.). The personal flow cytometer immunophenotype counts were indistinguishable from those of conventional flow cytometers for both peripheral blood samples (n=13; r=0.95; p<0.0001; bias of -1.1%) and lymph node aspirates (n=11,r=0.98; p<0.001; bias of 1%). All but one leukemic and one lymphomatous lymph node sample, out of 26 samples of dogs with lymphoproliferative disease analyzed, could be immunophenotyped as either B or T cells.

**Conclusions:**

We conclude that use of only 2 monoclonal antibodies is sufficient for immunophenotyping most cases of canine lymphoma by flow cytometry and enables rapid immunophenotyping. The personal flow cytometer may be as effectively used for immunophenotyping canine lymphoma as conventional flow cytometers. However, the personal flow cytometer is more accessible and user-friendly, and requires lower sample volumes.

## Background

Lymphoma is one of the most prevalent cancers in dogs [1]. Diagnostic testing and prognosis is based on clinical signs and degree of spread, morphological features of the lymph node and lymphocytes, and other cytopathologic features such as mitotic rate, and clonality of antigen-receptor rearrangement or of cluster of differentiation (CD) antigens.

Immunophenotyping CD antigens has contributed significantly to both diagnosis and prognosis of lymphoid neoplasia. This approach measures the binding of labelled, monoclonal antibodies to specific intracellular or surface CD antigens. It is well-established and has long been used in cell analysis, particularly in the fields of haematology and immunology [2-4]. For lymphoma, it can be accomplished using either immunohistochemistry of tissue-biopsy sections [5] or by immunocytochemistry of fine needle aspirates. Cytologic analysis can be done manually on smears using microscopy [6] or on cell suspensions using automated, flow cytometry.

Immunophenotyping is most easily and rapidly accomplished by flow cytometry. Flow cytometry of blood, lymph node and bone marrow samples may improve evaluation and prognosis of dogs with lymphoma [4,7-9]. However, in veterinary medicine this technique is mainly available only as a research tool, rather than for widespread diagnostic use as occurs in human medicine [9]. There are only a few European laboratories that routinely provide immunophenotyping by flow cytometry for veterinary patients. The main barriers associated with the expansion of flow cytometry in veterinary medicine are the substantial cost of the analyser, reagents, and facilities, and the need for advanced training of the instrument operators. In addition, analysis and interpretation of results requires understanding and knowledge of flow cytometry and its principles.

It is well documented that immunophenotype of neoplastic lymphocytes correlates significantly with the survival time of dogs with lymphoma and is of significant value in prognosis [10-15]. In 175 dogs with lymphomas, T-cell phenotype had shorter relapse-free time (52 versus 160 days, p<0.001) and shorter survival times (153 versus 330 days, p<0.001) than B-cell phenotype [15]. Dobson, Blackwood et al. 2001 found that the T- cell phenotype is associated with a significantly shorter recurrence-free interval and reduced survival times. Hazard ratio for T-cell versus B-cell immunophenotype lymphoma was 4, with 95% confidence interval from 1.4 to 11.3, p=0.035. However, the correlation of immunophenotype with prognosis is not perfect, and exceptions have been identified. For example, the small and clear T-cell lymphoma has one of the best prognoses [11]. Thus for prognosis, immunophenotyping data must be used along with other clinical and cytological assessments of degree of spread, morphologic features, and mitotic rate.

In recent years, multiple research studies involving immunophenotyping by flow cytometry have been published in the veterinary literature [2-4,6,15-20]. However, limited veterinary availability of flow cytometers, expertise, and reagents is a major limitation to the diagnostic use of immunophenotyping [9]. Also, the current protocols used in veterinary diagnostics are labour intensive and time-consuming, involve standard technology, and have not been customised for widespread diagnostic use in the clinical pathology laboratory.

The recent development of the personal flow cytometer may overcome the barriers previously associated with diagnostic use of flow cytometry in veterinary medicine. The personal flow cytometer is a miniaturised, user-friendly, and affordable version of the standard instrument [21] found in dedicated facilities and operated by dedicated personnel. Different manufacturers name the instruments they produce “personal”. Such instruments include the different Guava platforms produced by Millipore, the Accuri C6 from BD Biosciences, the Attune flow cytometer by Applied Biosystems and the HPC-100 system by Handyem. The technical bulletin for the Accuri C6 analyzer from BD Biosciences is entitled “Making Flow Cytometry Personal”[22] They can be used in any laboratory and by any laboratory technologist, and seem ideal for the veterinary clinical pathology laboratory.

The Guava flow cytometer (Guava Technologies, Hayward, CA, US) is the first, such personal flow cytometer, and has several advantageous features for veterinary use. It is an automated, easy-to-use, bench-top, single-cell, analyser that can perform a wide range of multi-parameter, cell-based assays using light scatter and multiple fluorescence measurements and also direct measurement of cell counts without the need for reference beads [23]. It requires far less sample and reagents volume [24], making it ideal for veterinary and diagnostic cytology applications. It requires far less operator time and expertise, less maintenance and is automated for multiple tube or microtiter plate sampling. It requires far less bench space than the standard analyser. The purchase cost is considerably lower than conventional flow cytometers. Also, it does not produce voluminous waste, because it does not use sheath fluid to move the cells through the analyser [21].

The aim of this study was to adapt the immunochemical protocol used for immunophenotyping of canine lymphoma to the personal flow cytometer for rapid, effective and user-friendly application to diagnosis and prognosis of canine lymphoma and to demonstrate its practicality for widespread veterinary application. Parts of this study have also been presented in the 10^th^ and 13^th^ Annual Congresses of the European Society of Veterinary Clinical Pathology in Barcelona, Spain and Dublin, Ireland [25,26].

## Methods

### Subjects

Samples from 58 dogs were obtained: a) 21 peripheral blood samples (of which 1 was leukemic) were used to assess the ability of the personal flow cytometer to effectively discriminate lymphocytes from other leukocytes, based on forward and side light scatter (4 of these were also immunophenotyped). The non-leukemic samples were obtained from non-lymphopenic dogs that presented to the University Veterinary Hospital in Dublin for a variety of reasons other than lymphoma/leukaemia. b) 41 samples were used to assess the ability of the personal flow cytometer to immunophenotype: 23 were lymph node aspirates from dogs with lymphoma, 2 were lymph node aspirates from lymphoma-free dogs, 13 were non-leukemic peripheral blood samples and 3 were leukemic blood samples. Of the 41 samples, immunophenotyping results for 25 (14 peripheral bloods and 11 lymph node aspirates) were compared between the personal flow cytometer and the conventional analyzers, and 16 were analyzed on the Guava only as no other analyzer was available. All investigations were undertaken within the guidelines of the UCD Animal Research and Ethics Committee.

### Flow cytometry

#### Instrumentation

The Guava EasyCyte Plus is equipped with a 20 mW, argon-ion laser of 488 nm, a forward-scatter (FSC) detector, a side-scatter (SSC) detector, and four fluorescence detectors (525/30 nm, 583/26 nm, 680/30 nm, and 785/70 nm). The Cyan Dako was equipped with three lasers (488 nm, 635 nm and 405 nm) and the BD FACSCalibur with 2 lasers operating at 488 and 635 nm. The Guava ExpressPro software program (version 5.0) was used for sample data acquisition on the Guava, and the dedicated software for the other analyzers (Dako Summit and BD FACStation respectively).

#### Data acquisition

Gating strategy and protocol setup was the same for all instruments. When acquiring data, a lymphocyte gate was defined, based on the size and granularity of lymphocytes depicted on the forward scatter versus side scatter plot. A debris threshold was set to eliminate events having low forward and side scatter properties. The gains for forward and side scatter were set to allow good visualization of the different cell populations contained in the sample, as well as to give adequate separation of groups on the cytogram. This was useful in order to apply the lymphocyte gate correctly. In lymph node aspirates, the same principle was followed to set the debris threshold, but the need for subpopulation separation was minimal due to the more homogeneous population of lymphocytes and the high lymphocyte yield obtained from these samples. A total of 5,000–20,000 cells within the lymphocyte gate were counted from each sample. Samples were analyzed together with negative isotype controls (AbD Serotec Canine Negative Controls/Isotypes for CD3 and CD21, Oxford, UK, conjugated to the same fluorochromes as the monoclonal antibodies) to allow the accurate establishment of the limits of fluorescence and exclude fluorescence caused by auto-fluorescence and by non-specific binding. To avoid contamination of FITC fluorescence into the PE channel, fluorescence compensation was set by examining separately sample lymphocytes stained with anti-CD3+FITC antibodies and sample lymphocytes stained with anti-CD21+PE antibodies (no standard controls were used to set compensation). Compensation factor for the FITC channel was 9–11% and for the PE channel was 0–0.3%. Forward scatter gain was set at 16x and 100%. Side scatter gain was set between 475–485 V. For the FITC channel (GRN), the gain was set at 600–700 V and for the PE channel (YLW), at 500–600 V.

#### Quality control

Prior to acquisition, the Guava’s performance was assessed by using the Guava Easy Check kit (Millipore). The kit is composed of beads and a diluent. According to manufacturer’s recommendations, a fresh solution of beads was prepared and run in triplicate daily. The average number of particles/ml, the average FSC and SSC intensities, and the average fluorescence intensities for green, yellow, red and near infra-red channels were calculated, along with the coefficients of variation (CV%) for these parameters. If they fell within the range proposed by the manufacturer (less than 10% for particle counts, and <5% for fluorescence intensities) sample analysis was performed. The daily quality control and maintenance for the reference instruments were performed by dedicated personnel in the core flow cytometry facility used.

#### Evaluation of guava effectiveness in lymphocyte identification

In peripheral blood samples no lymphocyte isolation techniques were applied and no non-lymphocytic markers were used. Accordingly, it was necessary to demonstrate that the gate set around events consistent with lymphocytes (based on their forward and side scatter characteristics) effectively discriminated them from other leukocytes. Guava lymphocyte percentages for 21 (20 non-leukemic and non-lymphopenic peripheral blood samples, and 1 leukemic) were compared with those obtained by the conventional, automated haematology analysers (n=11 on the CELL DYN 3500 and n=10 on the ADVIA 2120).

#### Immunophenotyping

##### Peripheral blood samples

Canine peripheral blood samples were collected into potassium EDTA (Sarstedt, Wexford Ireland). The samples were submitted to the veterinary clinical pathology laboratories at University College Dublin, University California Davis Veterinary Teaching Hospital, or Central Diagnostic Services, Cambridge, UK, between May 2008 and December 2011. All blood samples were initially processed with a haematology analyser; also blood smears were stained with a Romanowsky-type stain (Aerospray 7120, Wescor, Accu-Science) and a manual differential count was performed.

#### Lymph node aspirates

Lymph node samples were obtained by two different ways: a) for cytological examination, 21 gauge hypodermic needles were used (non-aspiration technique, needle insertion and redirection without applying negative pressure). The cells were immediately expelled on glass slides using a 5 ml disposable plastic syringe and smears were prepared, air dried and stained with a Romanowsky-type stain (Aerospray 7120, Wescor, Accu-Science, Dublin) b) for flow cytometric examination, 2.5 ml disposable syringes prefilled with 1 ml of sterile phosphate-buffered saline (PBS) at pH=7.4 and 21 gauge hypodermic needles were used. The needle was attached on the syringe and inserted multiple times in the lymph node while applying negative pressure. Once the fluid became opaque (occasionally blood-tinged), the needle was removed from the lymph node and the fluid was expelled (with the needle removed from the syringe) in an EDTA container. Lymph node aspirates were examined by the conventional haematology analysers (CELL DYN 3500 and ADVIA 2120) to assess their nucleated cell count. Cytospin preparations (Cytospin 4, Thermoshandon, Thermo Scientific Kalamazoo, MI, USA) were occasionally obtained from the lymph node aspirates prior to analysis, stained and examined by optical microscopy to assess sufficient lymphocyte retrieval. If the vast majority of the nucleated cells were lymphocytes by qualitative assessment, analysis of the sample was considered possible.

#### Sample preparation and staining

Samples from the University Veterinary Hospital in Dublin were analyzed immediately after sampling. Lymph node aspirates from external practitioners were mixed 1:1 with cell preservative (Streck Cell Preservative, Streck, USA) and shipped occasionally on ice. They were initially filtered (Partec, CellTrics, 50 μm filters) and a cell count was performed on the automated haematology analyzers to assess the sample cellularity. Aspirates sent from external sources were first stained with propidium iodide (P4170, Sigma-Aldrich) to assess viability and were immunophenotyped only if cell viability was >90% to avoid non-specific staining. Lymphocytes for immunophenotyping were obtained from 100 micro liters (μL) of blood; for lymph node samples a volume containing 0.5-1×10^6^cells was used based on the previously performed cell count; direct staining was performed with murine, anti-canine undiluted monoclonal antibodies added according to the manufacturer’s recommendations, without being previously titrated: a) anti-CD3 conjugated with fluorescein isothiocyanate (source AbD Serotec CA17.2A12), or Alexa Fluor 488 (Professor P.F. Moore, University California, Davis, USA CA17.2A12-IgG-1) (excitation=494 nm, emission=518 nm), and b) anti-CD21 conjugated with phycoerythrin (PE, excitation=488 nm, emission=667 nm, Professor P.F. Moore, University California, Davis, USA CA2.ID6-IgG-1). The cell suspension was placed in dark for 30 minutes at room temperature. After staining with the antibodies, the erythrocytes were lysed if needed (for blood samples and only for grossly blood contaminated lymph node aspirates) by adding 900 μL of red cell lysing solution (Guava Lyse, Guava) to a final volume of 1 ml. The samples were left in dark for 10–15 minutes at room temperature and then spun down at 2000 rpm (~200 g, Spectrafuge 16 M microcentrifuge, Labnet international) for 5 minutes. If the pellet’s colour was red, the lysing step was repeated. After red cell lysis, the nucleated cells were washed twice with phosphate-buffered saline (PBS) at pH=7.4 containing 3% foetal calf serum. Finally, the cell suspension was diluted with washing buffer and the cell concentration was adjusted to less than 500 cells/μL. In most cases, the exact same samples were analyzed on the personal and the conventional flow cytometers.

#### Fluorescence microscopy

Two lymphoma lymph node aspirates (B-cell and T-cell) and one non-cancerous peripheral blood sample previously stained for immunophenotyping were additionally examined by fluorescence microscopy in order to assess the cell type and cell percentage stained, and the sub-cellular distribution and quality of staining with the monoclonal antibodies. After being analysed on the personal flow cytometer, the cells were added into a 96-well plate with a final concentration of 50,000 to 100,000 cells in 100–200 μL of phosphate buffer saline per well. They were then incubated with Hoechst (nuclear) stain (Hoechst 33342, Invitrogen): 1.5 μl of a 1 mM solution was diluted in 1 ml of phosphate buffered saline pH=7.4 and 100 μl of the final solution was added to each well, giving a final volume of 200–300 μl. The cells were left incubating for 15 minutes at room temperature in the dark. They were examined using the InCell 1000 analyzer (GE healthcare, Cardiff, UK). The excitation/emission filters were set as follows: a) Hoechst channel (nuclei): excitation 360 nm, emission 460 nm b) FITC channel (CD3+): excitation 475 nm, emission 535 nm and c) PE channel (CD21+): excitation 570 nm, emission 620 nm.

#### Statistics

Statistical analysis was performed on Graph Pad Prism version 5.0 (LaJolla, USA). The data was first checked for normality using D’Agostino and Pearson’s omnibus normality test (α=0.05). Parametric tests (Pearson’s r) were used for assessing correlation in normally distributed data sets and non-parametric tests (Spearman’s r) for data that were not normally distributed. No attempt was made to achieve normality. Bias and agreement were calculated through Bland-Altman analysis. Correlation was characterized as excellent (r≥0.93), good (r=0.80 to 0.92), fair (r=0.59 to .79) or poor (r ≤0.59) [27,28]. Linear regression analysis was also performed. The significance of differences between the means or the medians were assessed through paired tests for normally (Paired t-test) and not normally (Wilcoxon matched pairs test) distributed data. When values from a particular sample in a data set were more than three standard deviations away from the mean of the rest of them, the sample was considered an outlier and was removed from the data set [29]. The immunophenotyping results for blood and lymph node samples were analysed separately and collectively.

## Results

**Figure 1:**
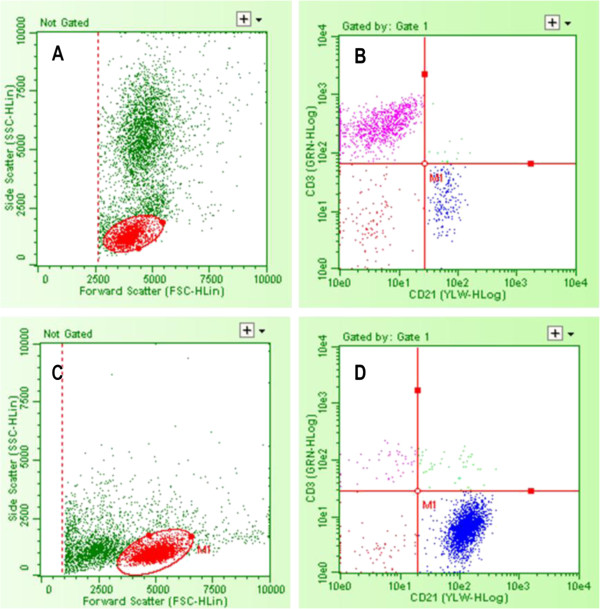
**Scatterplots of immunophenotyping data from the personal flow cytometer for normal blood and a lymphomatous lymph node.** (**A**) Non- leukemic peripheral blood with debris threshold set on forward scatter, oval lymphocyte gate and population of granulocytes, (**B**) Non-lymphoma peripheral blood immunophenotypes: 78% of the cells contained in the lymphocyte gate are CD3+ and 12.6% CD21+, the rest being mostly unstained events (**C**-**D**) B-cell lymphoma in a lymph node aspirate, 98.5% of the gated cells are CD21+.

**Figure 2:**
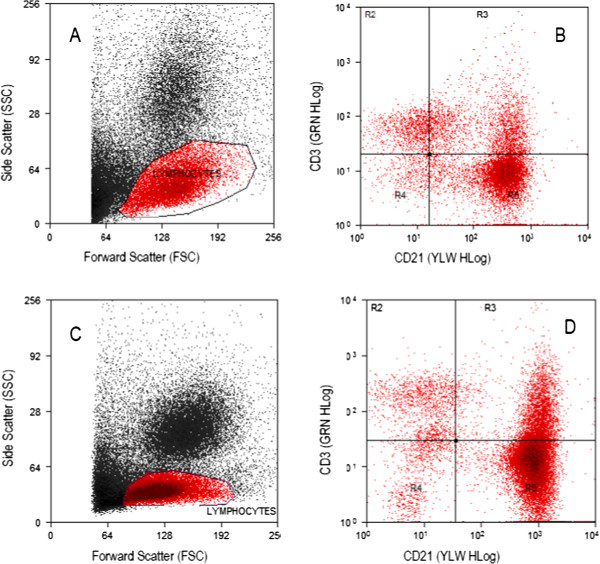
**Comparison of scatterplots of immunophenotyping data from the personal and conventional flow cytometers for leukemic blood.** Peripheral blood sample from a dog diagnosed with (CD 21+) large B-cell lymphoma based on clinical findings, lymph node cytology and lymph node immunophenotyping. The dog had a WBC count of 38.7×10^9^/L (ADVIA 2120) with 55% lymphocytes (21.3×10^9^/L). Predominance of (CD21+) lymphocytes (area R5 of the scatterplots). Scatterplots (**A**) and (**C**) show the total events as acquired by the personal and the conventional analyzer respectively (2 times more events were acquired by the conventional for this sample). The lymphocyte gate is showed containing 34% of the total events for the Guava and 53% for the conventional analyzer. Scatterplots (**B**) and (**D**) illustrate the immunophenotyping results: the Guava gave 64% (CD21+), 10.2% (CD3+), 21.7% double positive and 4.3% double negative. The conventional analyzer gave 71.7% (CD21+), 10.1% (CD3+), 13% double positive and 5.2% double negative events (Dako Summit v4.3 was used to analyze data).

The appearance of the personal cytometer scatter plot for a normal peripheral blood sample and a lymph node aspirate from a dog with lymphoma are depicted in Figure 1. There is adequate cell population separation in both the forward-scatter versus side scatter plots and the green fluorescence versus yellow fluorescence plots. For the control, non-lymphopenic peripheral blood samples that were used, there is good visualization of the lymphocyte population that facilitates their gating. Occasionally, there was an overlap between the lymphocytes and a cluster of events close to the lower end of both axes, and/or the cluster of events with higher forward-scatter and side-scatter values consistent with monocytes. A comparison between the scatter plots generated by the personal flow cytometer and a conventional analyzer (Cyan, Dako) for the same sample can be seen in Figure 2. The appearance of both the forward scatter versus side scatter and the fluorescence intensity plots is similar for both instruments.

### Guava effectiveness in lymphocyte identification

**Figure 3:**
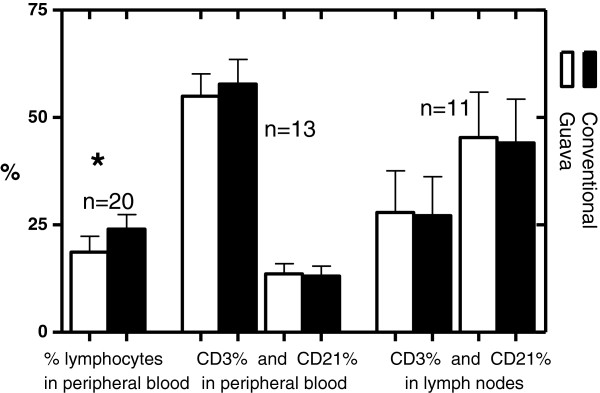
**Comparison of differential lymphocyte counts and immunophenotypes from the personal and conventional flow cytometers.** The bars illustrate the mean ± SD for the percentages obtained by the Guava and the conventional analyzers: differential lymphocyte percentage (peripheral blood), and (%) CD3+, (%) CD21+ for the blood samples and the lymph node aspirates. * Group differences significant at p < 0.001.

**Figure 4:**
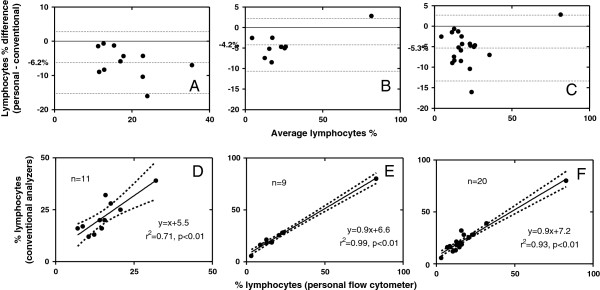
**Comparison of differential, blood, lymphocyte counts on the personal flow cytometer and automated haematology analysers: Bland-Altman bias and linear regression plots.** The mean bias (central dot-line) and limits of agreement (upper and lower dotted lines) are indicated in the bias plots and the regression line with 95% confidence interval for the regression plots. (**A** and **D**) % lymphocytes Guava versus CELL DYN 3500, (**B** and **E**) % lymphocytes Guava versus ADVIA 2120, (**C** and **F**) % lymphocytes Guava versus both analyzers.

21blood samples were examined for the differential lymphocyte counts in total. One sample had to be removed as an outlier because its value (expressed as the difference between the personal and the conventional analyzer) was 10 standard deviations away from the mean of the rest (also expressed as differences). Based on forward and side scatter parameters and the positioning of the lymphocyte gate, the mean lymphocyte percentage for peripheral blood samples on the Guava was 18.7%±16.5% (median 15.2%, range 3.1% – 82.8%) and on the haematology analyzers the mean was 24%±15.2% (median 20%, range 5.7% – 80%) (Figure 3). A Wilcoxon matched pairs test showed statistically significant difference between the medians (p=0.0002). The bias and linear regression plots are depicted in Figures 4A-4C and 4D-4F respectively. Correlation for lymphocyte percentages between the Guava and both automated haematology analyzers was excellent (r=0.97, p<0.0001). Between the Guava and the CELL DYN 3500 the correlation coefficient was good, r=0.82 (p=0.0033, n=11), and between the Guava and the ADVIA 2120 it was excellent, r=0.96 (p<0.0001, n=9). The Bland-Altman analysis revealed a mean negative bias for blood samples of -5.3% (SD of bias = 4%), and was similar between the Guava and the CELL DYN 3500 (-6.2%, SD of bias = 4.6%) and between the Guava and the ADVIA 2120 (-4.2%, SD of bias = 3.3%).

### Immunophenotyping

The sample-preparation protocol took approximately 1.5 to 2 hours to perform, from the point of receipt of sample to the point of starting analysis by the flow cytometer. The flow cytometric analysis took approximately 0.5 to 1 h from the point of starting the analysis to the point of reporting immunophenotypes.

**Figure 5:**
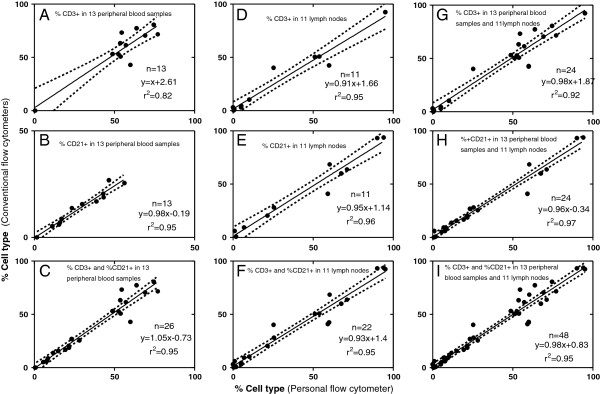
**Comparison of lymphocyte immunophenotypes in blood and lymph node aspirates determined on the personal and conventional flow cytometer: linear regression plots.** Results for cell types (%) obtained by the Guava and the conventional analyzers. Regression line and 95% confidence interval. (**A**-**C**) % CD3+, % CD21+ and collectively % CD3+ and % CD21+ in peripheral blood samples. (**D**-**F**) % CD3+, % CD21+, and collectively % CD3+ and % CD21+ in lymph node aspirates. (**G**-**I**) % CD3+, % CD21+ and collectively % CD3+ and % CD21+ in all peripheral blood and lymph node samples.

**Figure 6:**
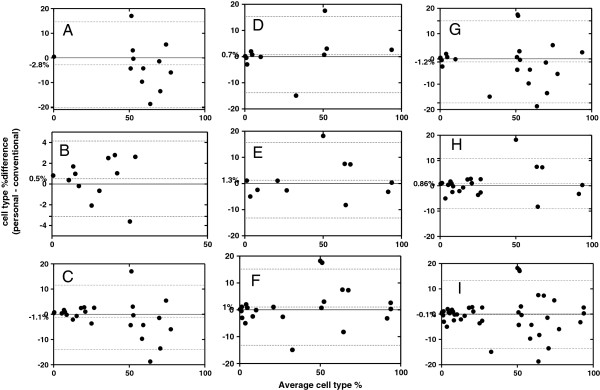
**Bland**-**Altman bias plots for immunophenotyping lymphocytes in blood and lymph node aspirates using the personal compared to the conventional flow cytometer.** Difference versus average for cell percentages. Mean bias (central dot-line) and limits of agreement (upper and lower dotted lines). (**A**-**C**) % CD3+, % CD21+ and % CD3+ and % CD21+ in peripheral blood samples. (**D**-**F**) % CD3+, % CD21+, and % CD3+ and % CD21+ in lymph node aspirates. (**G**-**I**) % CD3+, % CD21+ and % CD3+ and % CD21+ in all peripheral blood and lymph node samples collectively.

Linear regression and bias plots for immunophenotyping showed that the personal analyzer and the conventional analyzers gave similar results and were highly correlated for both blood and lymph node aspirates (Figures 5 and 6).

### Blood samples

14 samples were examined in total, but one sample had to be removed as an outlier since its value (expressed as the difference between the personal and conventional flow cytometer) was 15 standard deviations away from the mean of the rest (also expressed as differences). Coefficient of correlation was excellent (r=0.95, p<0.0001) when percentages for both CD3+ and CD21+ cells were assessed together. For CD3+ cells it was good(r=0.90, p<0.0001), and for CD21+ cells it was excellent (r=0.97, p<0.0001). The Guava showed a negative mean bias of -1.1% for blood immunophenotypes (SD of bias = 6.5%), which was negative for CD3+ cells (-2.8%, SD of bias=8.9%) and positive for CD21+ cells (0.5%, SD of bias=1.8%).

### Lymph node aspirates

11 lymph node aspirates were examined. Correlation was excellent for CD3+ (r=0.97, p<0.0001), CD21+ cells (r=0.98, p<0.0001) and also for CD3+ and CD21+ cells combined (r=0.98, p<0.0001) between the Guava and the conventional flow cytometers. The mean bias for the lymph node samples was 1% (SD of bias = 7.2%) with a positive bias for the different immunophenotypes (0.8%, SD=7.4% for CD3+ cells, and 1.3%, SD=7.3% for CD21+ cells).

Overall, for all the blood and lymph node samples (n=24) that were compared between the Guava and the conventional analyzers and for both CD3+ and CD21+ cells, the coefficient of correlation was excellent (r=0.96, p<0.0001, n=48) and the Guava showed a mean negative bias of -0.1% (SD of bias = 6.9%) (Figures 5I and 6I).

**Figure 7:**
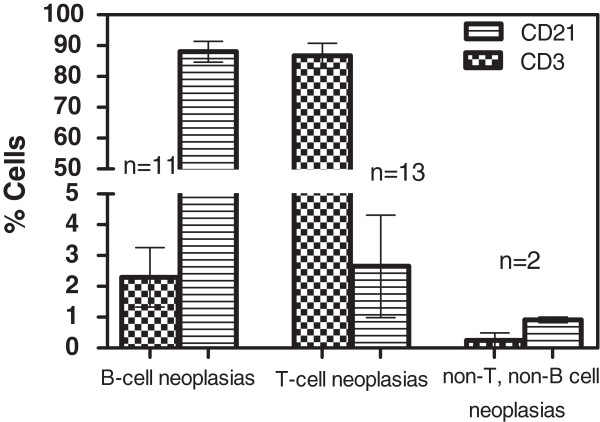
**T and B immunophenotype predominance in lymphoproliferative disease (23 lymphomas; 3 lymphocytic leukaemias).** (%) CD21+ and (%) CD3+ percentages obtained by the Guava analyzer for the lymphoma / leukaemia samples included in this study. The percentages are shown separately for the T-cell, the B-cell and the non-T non-B cell tumors. There is clear separation between the cell populations allowing the identification of the predominant immunophenotype in most cases.

With the 2 antibody protocol that was used, 24 out of 26 dogs (92%) with lymphoproliferative disease were successfully immunophenotyped (Figure 7). One lymph node aspirate from a dog with lymphoma and one case of acute lymphoblastic leukaemia were negative for both antibodies. The 24 tumors successfully immunophenotyped were 11 T-cell lymphomas, 11 B-cell lymphomas and 2 T-cell leukaemias. In most cases there was clear predominance of one cell type (≥50%) allowing the immunophenotype identification.

**Figure 8:**
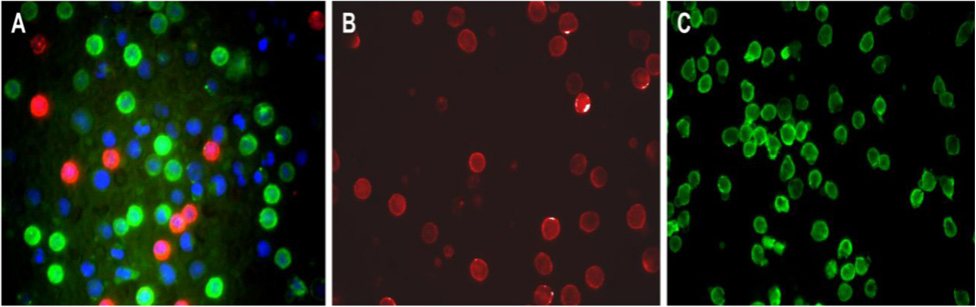
**Fluorescence microscopy images of immunophenotyped lymphocytes of blood and lymph nodes from a healthy dog and dogs with lymphoma.** Lymphocytes stained with anti-CD3+FITC (green color) and anti-CD21+PE (red color) antibodies and Hoechst nuclear stain (blue color), original magnification 40×. (**A**) Fused image (360 nm, 480 nm, 525 nm) of normal peripheral blood from a dog, predominance of (CD3+) T-lymphocytes. (**B**) B-cell lymphoma, lymph node aspirate (picture taken after excitement at 525 nm). All the cells seen are (CD21+) (**C**) T-cell lymphoma, lymph node aspirate (picture taken after excitement at 480 nm). All the cells seen are (CD3+) T-lymphocytes.

Fluorescence microscopy images revealed staining of most of the small mononuclear cells consistent with lymphocytes with no non-specific binding. The staining intensity was high, and the tagged antibodies were uniformly dispersed across the cell membranes with no intracellular localization (Figure 8).

## Discussion

### Novel features of immunophenotyping approach

This study demonstrates a simple and cost-effective approach to diagnostic application of flow cytometry in the clinical pathology laboratory for immunophenotyping lymphoma. It is based on an adaptation of a standard, immunohistochemistry protocol and the use of the personal flow cytometer.

The protocol is based on using the most simple strategy and assay for immunophenotyping of most canine lymphomas, that is, using single T and B cell surface markers. Intracellular markers for flow cytometry are more technically demanding and the evaluation panel is frequently restricted to easily accessible surface antigens only, although it has become evident that some lineage markers are already expressed in the cytoplasm very early in differentiation [30]. The surface panel approach simplifies the immunophenotyping assay, reduces assay time and thereby minimizes interference with other laboratory activities, and also reduces sample volume requirement and allows application when only small samples are available. In this study, it was effective for 92% of 26 lymphomas and lymphocytic leukaemias.

The personal flow cytometer is a scaled-down version of the conventional analyser, but with most of its functional capacity, and also with substantial technological differences [21]. It has numerous advantages for the clinical pathology laboratory over the conventional flow cytometer. The latter is usually located in a remote core facility, needs to be operated by dedicated, expert technologists, has multiple users, and requires pre-scheduling for use [9]. In contrast, the personal flow cytometer is easily and immediately accessible because its small size and lower bench-space requirement allow it to be situated directly in the clinical pathology lab. Also, it is generally less complicated to use and easier for clinical pathology technologists to learn a specific assay on [31]. Use of microcapillary technology results in lower volume requirements for both samples and reagents, thereby reducing assay cost as well as sample size [24,32,33]. In most cases in this study, the Guava used less than 100 μL per sample, whereas the conventional instruments consumed significantly more. Maintenance is reduced because of the elimination of sheath fluid which is used in conventional cytometers to cause the cells to flow past the laser beam. The smaller analyser size is associated with a lower cost of purchase, as well as low cost of maintenance and operation.

There were also technical advantages of the personal flow cytometer. The Guava provided absolute cell counts without a need for reference beads, which are frequently required by the conventional analysers [34]. Another desirable feature of the Guava is its automation. It is equipped with a plate holder for analyzing samples from 96-well plates, and can also hold and analyze samples from up to ten different tubes at once without user interference. The cleaning steps before, between and after sample analysis are performed automatically. We were able to solve any technical issues ourselves while using the analyzer. The flow cell is readily accessible and can be easily removed to be cleaned or substituted.

### Correlation and bias of personal versus conventional flow cytometers and automated haematology analysers

For cell identification and the determination of the percentage of lymphocytes in peripheral blood samples, there was good (r=0.80–0.92) to excellent (r≥0.93) correlation between the Guava and the automated haematology analyzers. Whole blood lysis results in samples containing a mixture of peripheral blood leukocytes, and a method for identifying the population of interest was required. We used the simplest approach of separating leukocytes depending on their forward scatter and side scatter characteristics, after setting a threshold to exclude cell debris. Alternatively, specific surface immunomarkers or density gradient separation for the isolation of mononuclear cells/lymphocytes could have been used [35], but that would add to the preparation and analysis time; it is reported that density gradient separation should probably not be used in samples containing abnormal lymphocytes, as they may have altered density and could be lost during preparation [36].

The difference plots for the lymphocyte percentages indicate that there is a small negative bias (-5.3%) between the Guava and the conventional analyzers. The underestimation of lymphocyte percentages by the Guava is similar for both conventional analyzers. The haematology analyzers used are designed for and specialize in performing differential cell counts for peripheral blood samples. The CELL DYN 3500 uses optical and impedance methodology, while the ADVIA 2120 also uses cytochemical staining to discriminate between the different cell types. The sample processing before the differential count was different for the two methods, as the samples analyzed on the Guava had to undergo manual red cell lysis and then centrifugation and washes. It is possible that there was a loss of lymphocytes during the preparation procedure. Also, the monocytes were not always readily identified as a separate population, and the gating had to be tightly set around lymphocytes to avoid overlap with other cell populations. This probably also contributed to the lower percentage of lymphocytes detected by the Guava compared to the haematology analyzers.

Our finding of cost-effective, accurate and rapid immunophenotyping of canine lymphoma using the personal flow cytometer is similar to the findings of other studies on immunophenotyping lymphocytes in HIV infection in humans [24,31,33,34,37,38]. These studies used a similar approach by assessing correlation and agreement with linear regression and Bland-Altman bias analysis. For immunophenotyping of lymphocytes, the correlation for T-cell and B-cell percentages was excellent for the blood samples and the lymph node aspirates (r=0.95 and r=0.98 respectively, p<0.0001). In the difference plots, there is a slight mean bias that is negative for blood samples (-1.1%) and positive for lymph node aspirates (1%). The bias variation increases for higher measurements, with similar distribution across the line of identity. Since, in this study, the purpose is to identify which cell population shows marked predominance compared to the other, in order to identify the predominant immunophenotype, the magnitude of this bias is unlikely to affect the final outcome.

### Effectiveness of 2-antibody approach for immunophenotyping

A total of 41 samples were immunophenotyped (26 from dogs with lymphoproliferative disease, and 15 disease-free dogs) and 2/41 samples did not stain with either of the antibodies used. All 13 disease-free dogs and 24 out of 26 cases (92%) with lymphoproliferative disease were successfully immunophenotyped. In most of the latter, there is a clear predominance of one immunophenotype compared to the other, and the tumour was classified according to the predominant cell type. It has been reported that a cut off of 60% can be used to classify the tumour as B- or T-cell type [39], and that in human medicine a cut off of >20% of the gated cells is used to consider a marker expressed, but no specific guidelines for veterinary medicine have been reported[4]. In our cases, the predominant cell type (in the tumors that were not negative for both markers) was ≥60% of the gated cells in all samples but one lymph node aspirate, in which ~50% of the gated cells were CD3 positive and ~7% CD21positive. Based on the predominance of T-cells and the cytological diagnosis of lymphoma, the tumour was considered to be of the T-cell phenotype.

Both antibodies in this study were surface markers. CD3 is considered a pan T-cell marker expressed in early thymocytes and throughout the maturation of the cell [39]. CD21 is a surface marker expressed on mature B cells [39], but is frequently used due to its convenience. We have used the intracytoplasmic CD79a in the past, but found that it significantly prolongs the sample preparation time and is more technically demanding. Two-antibody immunophenotyping is frequently used in immunohistochemistry / immunocytochemistry [5,6] and is likely to cover the majority of the cases encountered in routine practice. The approach that we followed for the use of antibodies was the simplest possible. The amount of antibody solution used per test was provided by the manufacturers. A titration study was not performed prior to use, although no significant differences in immunophenotyping results were seen when serially diluted antibody solutions (up to 1:10 with PBS) were used for non lymphoma / leukaemia blood samples (data not shown). However, the same may not apply to cancerous samples as surface receptor expression may be altered.

Immunophenotyping (whether performed on the personal and the conventional machines or the personal flow cytometer only) revealed approximately equal numbers of B-cell and T-cell tumors. Most studies in canines typically show a predominance of the B-cell type [5,8,15,17,18,20], although a mild, T-cell lymphoma predominance was reported in one study [11]. One double negative lymph node aspirate and one leukemic blood sample were identified, as has also been described in other studies [5,15] but no other reported aberrant types described [39,40] were encountered.

### Fluorescence microscopy

The fluorescence microscopy images proved useful in order to assess the quality and distribution of staining. As expected by using surface markers, the tagged antibodies were localized across the cell membranes. The majority of the small mononuclear cells consistent with lymphocytes for each field of view were stained with no non-specific or intracellular binding. The predominant cell types in the fluorescence images were in accordance to the predominant cell types obtained by flow cytometry.

### Limitations of the study

There are several limitations of this study that should be considered when interpreting the results.

### Choice of antibodies

Only two, monoclonal, species-specific, surface markers were used in this study although cross-reacting antibodies from other species have frequently been used in other studies [5,18,20]. Flow cytometric studies of immunophenotypes in canine lymphoma commonly use an extensive panel of surface and occasionally intracellular markers [4,16,18,20,35,39,41]. Our purpose was to minimize complexity and difficulty, and develop a widely-accessible working protocol for diagnostic use that would cover the majority of tumors (in our case 92% were successfully immunophenotyped). Investigation and understanding of the complex pathophysiologic mechanisms involved in the development and spread of canine lymphoma, requires an extensive antibody panel.

### Prognostic value of T versus B staining

Cytological examination of all the lymphoma cases presented in this study, revealed a predominance of medium and large lymphocytes [42-44], and the mitotic rate was estimated on the cytological specimens as has been described before [3]. No follow-up study was performed to assess the association between the immunophenotype and the disease-free intervals and survival times. However, these have been previously described [10-14]. The morphological, immunophenotypic, clinicopathological and clinical information should be assessed collectively to provide more prognostic information.

### Number of samples

The Clinical and Laboratory Standards Institute (CLSI) recommends at least 40 samples to be analyzed in a full, comprehensive method comparison study in which both accuracy and precision are assessed [45]. However, this is a partial comparison study in which we mainly focus on correlation and precision. The calculated sample size needed to detect a relevant correlation (r>0.7) with a specified significance level (α=0.05) and power (1-β=0.95) is at least 20 samples [46,47]. The number of samples for cross-comparison of lymphocyte percentages and immunophenotypes in this study meet this criterion (21 and 25 respectively). The limited number of samples reflects the caseload of our laboratory as well as the limited request of immunophenotyping by the veterinary practitioners. An attempt was made to increase the numbers by analysing samples from the University California Davis Veterinary Teaching Hospital and Central Diagnostic Services, Cambridge, UK and also by analysing blood samples from lymphoma/leukaemia free dogs.

### Use of haematology analyzers for lymph node aspirate counts

The automated hematology analyzers were used to assess the cellularity of the sample upon receiving it. If the cell count was low, the sample was considered unsuitable and discarded or, if possible, the sampling was repeated. The cell count obtained by the analyzer was used to adjust the cellularity prior to staining and acquisition. We consider acquiring cell counts for lymph node aspirates by haematology analyzers a reasonable approach since these machines have been validated and used extensively for performing cell counts in a variety of fluids other than blood in both human and veterinary medicine [48-50].

### Sample viability during storage and transport

External peripheral blood samples were shipped to our laboratory in EDTA containers, and lymph node aspirates in tubes with cell-preservative as described in the materials and methods section. Cell preservatives have been evaluated and found to maintain the integrity of white cells for at least 7 days [51,52], although in our case the samples were analyzed within 24 hours of sample collection. Blood and lymph node aspirates sent with no ice packs occasionally showed low viability in propidium iodide staining and had to be discarded. The reason for the low viability cannot be easily identified and may also be related to the sampling technique. No significant differences in the immunophenotypes of samples analyzed immediately versus those stored at 4°C for12-16 h was found in one study [35].

## Conclusion

This is the first report of a stream-lined, user-friendly, cost-effective immunophenotyping strategy for immunophenotyping canine lymphoma by flow cytometry in the veterinary, clinical pathology laboratory. We demonstrate that the simplest technical approach is inexpensive, rapid and highly effective at immunophenotyping most lymphomas in the dog. We also demonstrate that the personal flow cytometer (Guava EasyCyte Plus) is more suitable than the conventional flow cytometer for the clinical pathology lab for routine diagnostics.

## Competing interests

The authors declare that they have no competing interests.

## Authors’ contributions

The study's conception and design was done by PO'B, IB and SP. SP and IB carried out the experiments, the data collection and statistical analyses. AL provided technical assistance. EJ O’N provided scientific support and assistance in the organization of sample collection. PO’B supervised the laboratory work and contributed to the writing of the manuscript. All authors read and approved the final manuscript.
